# Application of TEM and HDRM in hydrogeophysical surveys in Meisibulake coal mine

**DOI:** 10.1038/s41598-022-25526-2

**Published:** 2022-12-09

**Authors:** Yanlong Zhang, Yanqing Wu, Haize Zheng, Yangcheng Xu, Xiaoyang Cheng

**Affiliations:** 1 School of Resources and Safety Engineering, Chongqing University, Chongqing, 400044 China; 2 Chongqing Construction Engineering Quality Supervision and Testing Center, Chongqing, 401147 China; 3 CCTEG Chongqing Research Institue, Chongqing, 400037 China

**Keywords:** Environmental sciences, Engineering

## Abstract

Water disaster is one of the major disasters threatening the safety production of coal mine, which is rank only second to gas disaster. And Meisbluke Coal Mine is seriously affected by water disasters. In order to find out the scope and location of water-bearing areas in Meisbluke Coal Mine. The comprehensive geophysical exploration method combining transient electromagnetic method (TEM) and high-density resistivity method (HDRM) is used to carry out this research. Firstly, the measuring area is determined and the relevant measuring points are arranged, and 73 TEM survey lines and 10 HDRM survey lines were arranged according to the requirements. Then, the principle, data processing method and main parameters of TEM and HDRM are introduced. The TEM detection results show that the thickness of Quaternary inferred by TEM is consistent with the geological conditions revealed by boreholes, and the thickness is about 50–80 m. And the water enrichment of the bedrock is obviously recharged by the Quaternary aquifer. Besides, the water-enriched areas in each elevation are marked and the water inrush runoff channel is deduced based on the 3D scenario inverted by TEM. And the detection results of the water-bearing areas by the two methods are in good agreement with each other, which can confirm and complement each other, and the interpretation of the data is scientific and reasonable with high reliability. Besides, the detection depth of HDRM is larger than that of TEM.

## Introduction

From the perspective of coal mine production accidents, water hazards are the second most serious threat after gas accidents^1–3^. Ascertaining the hydrological situation and the exact location of water source is the fundamental work of advanced water exploration in coal mine. The conventional survey method is to detect the core by arranging more boreholes^4^. This method is essential to determine the location and extent of goaf and water-bearing areas in coal mines. However, the implementation of drilling often costs a lot of time and money, especially for some projects under construction. Compared with the borehole detection method, the geophysical method is widely used in detection because of its high efficiency and nondestructive characteristics^5–7^.

Transient electromagnetic method (TEM) is characterized by high sensibility to bodies of low resistivity, higher transverse resolution (it can resolve water-conducting structures of over 400 m deep and dozen m transversely wide) and fast operation. It can rapidly and accurately accomplish detection to locate water-inrushing structures^8–10^. Chen et al.^11^ propose a novel transient electromagnetic method configuration, short-offset transient electromagnetic method (SOTEM), which not only improves the accuracy, but also enlarges the exploration depth for detecting water-enriched areas in coal mines ranging from 1000 to 1200 m depth. The grounded electrical source airborne transient electromagnetic (GREATEM) system can provide considerable prospecting depth, lateral resolution, and high detection efficiency. To verify its feasibility in goaf water, a field electromagnetic survey over Qinshui coal mine (Shanxi province, China) was conducted by Li et al.^12^, and the resistivity profile of survey area is clearly presented, which is consistent with the information provided by Shanxi Coal Geology Geophysical Surveying Exploration Institute, China. The result shows that the application of GREATEM system is an effective technique for resistivity detection of goaf water. Chang et al.^13^ established whole-space geoelectric model based on actual coal-measure strata data, and modeled the whole-space TEM response of the water-filled goaf using the finite-difference time-domain method. The results showed that the low-resistance areas of the resistivity contours can accurately reflect the water abundance of the mining goaf. The underground TEM was used to detect the water abundance of the mining goaf in a mine environment and its detection results were consistent with the actual results. Zhou et al.^14^ developed a transient electromagnetic disturbance (TED) testing system with an adjustable direction of polarization to compensate for the low-frequency performance, which is of significant value in developing a large-scale system-level TED testing system in the future. Yu et al.^15^ used large-loop transient electromagnetic method to detect the water-filled mined-out area. And the GDP-32 instrument and ANT-6 magnetic probe were utilized for the practical geological exploration. The device operated at a fundamental frequency of 16 Hz with the 120 V transmitter voltage and 15 A transmitting current. The inversion results suggested that the mined-out areas enriched by water always exhibit a very low-resistivity, the resistivity contour present closed circle sharp. Fan et al.^16^ firstly used multi-window time-sweep wavefield inverse transformation technology to convert the TEM data into the multiresolution pseudo-wave, then utilize the joint interpretation method to obtain the underground electrical distribution characteristics, the location of the water-filled zones and stratum's continuous undulations.

Besides, another geophysical exploration method-high-density resistivity method (HDRM) is also frequently used in advanced water detection, goaf location and etc. in coal mine. To study the feasibility of HDRM in detecting uncovering water-bearing structures in drift, three devices including Wenner, Dipole–Dipole and Schlumberger were implemented by Ma et al.^17^ to detect water-bearing structures below 635 m measuring line in selected drift. The detection results show well agreement with the conclusions of field hydrogeology surveys. Due to the complex geological structure and the unclear water-abundance in working face cross heading driving along faults in Yuncheng coal mine, Zhai et al.^18^ used ahead exploring water with three-dimensional DC resistivity technology and WGMD-4 high density electrical system, inversion with least squares method based on inequality constraints, establishing mathematics model using detection data adopted loop iteration and smooth filtration factor, predict the space position of underground water. Shi et al.^19^ put forward to effectively release the roof water with roadway drilling according to the three-dimensional HDR method detection results during the process of working stope mining, because the roof strata can be destroyed under the action of ground pressure, which makes the hydraulic relationship between roof aquifers better. The results show that three-dimensional HDRM is reliable for roof water advanced detection during working stope mining, which can effectively guide the roof water in sandstone to be released through roadway drilling. Li et al.^20^ proposed a three-dimensional (3D) induced polarization method characterized with multi-electrode array, and introduce it into tunnels and mines combining with real-time monitoring with time-lapse inversion and cross-hole resistivity method.

On March 26, 2017, the roof water inrush occurred in the east of + 1818 m A7 working face in Mesbulak Coal Mine. Since the water drainage capacity of the coal mine is inadequate, the shaft bottom was flooded, and the maximum water inflow volume was estimated to be 320 m^3^/h, which lasts for 7 days. In order to conduct scientific treatment of water disasters, the geophysical methods are used to quickly and effectively ascertain the cause of the roof water inrush and water supply channel of + 1818 mA7 east working face.

## Engineering background

### Overview

**Figure 1:**
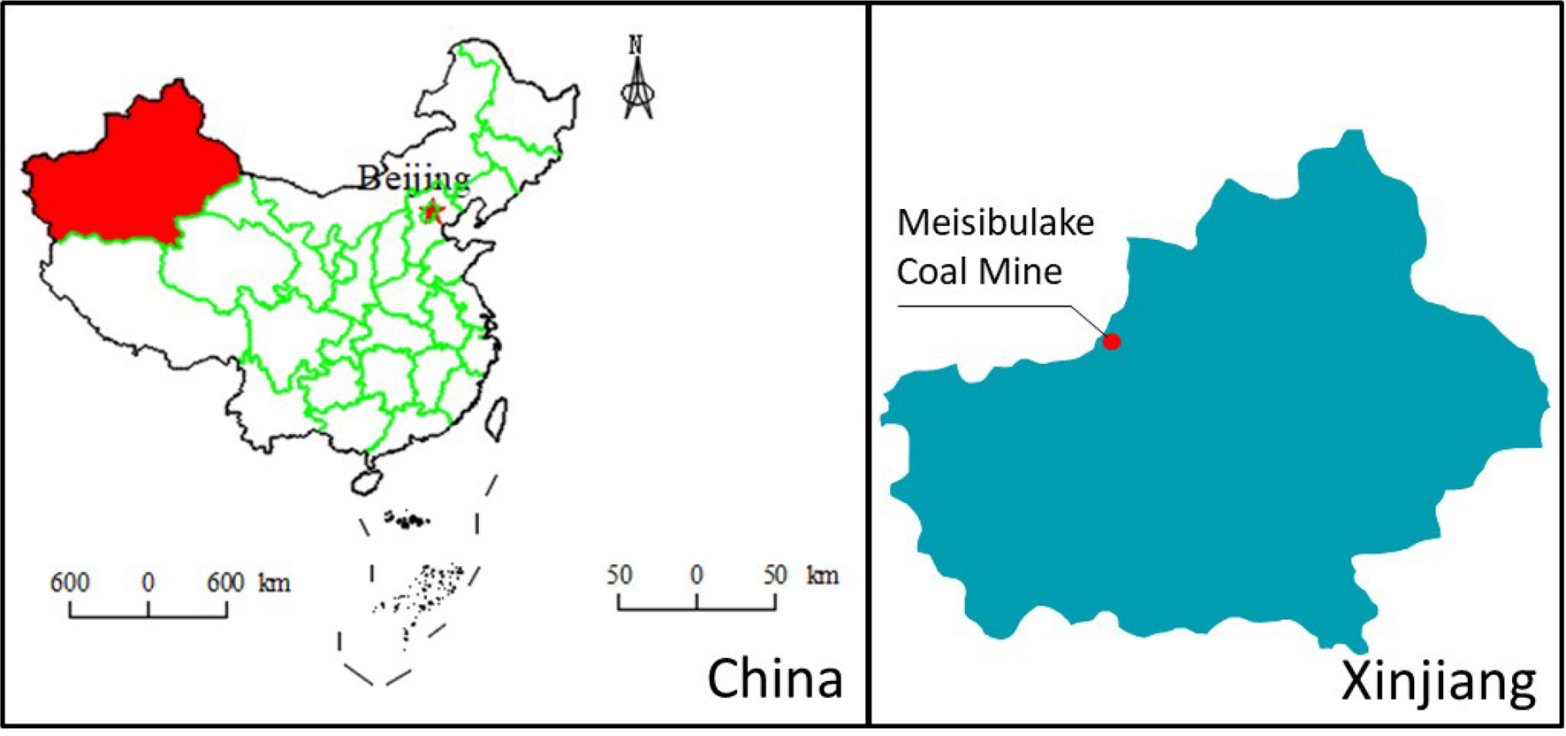
Study site.

Meisibulake Coal Mine of Baicheng County is located in Meisibulake Village (70 km northeast of Baicheng County) of Meisibulake River area, the administrative division of which is under the jurisdiction of Baicheng County of Akesu Prefecture, as shown in Fig. 1. The coal seam in the mining area is a steeply inclined with a dip angle of 61–72°. The occurrence of coal seam is stable, with a total of 6 exploitable coal seams, including 3 thin and medium thick coal seams and 3 thick coal seams (the maximum average thickness of coal seam is 7.6 m). The main mining coal seams are A3, A5, A6, A7, A8 and A9. The designed production is 600,000 t/a.

There were 2 abandoned coal mines in Meisibulake coal mine field. During the driving period of the 1910A3-1 return airway of 1 W (A3-1) 01 working face, it passed through the west abandoned coal mine in 2011. After that, the connected place was permanently sealed immediately, and the water was discharged from all the boreholes. Until February 21, 2011, a total of 258.09 m^3^ of water was released.

**Figure 2:**
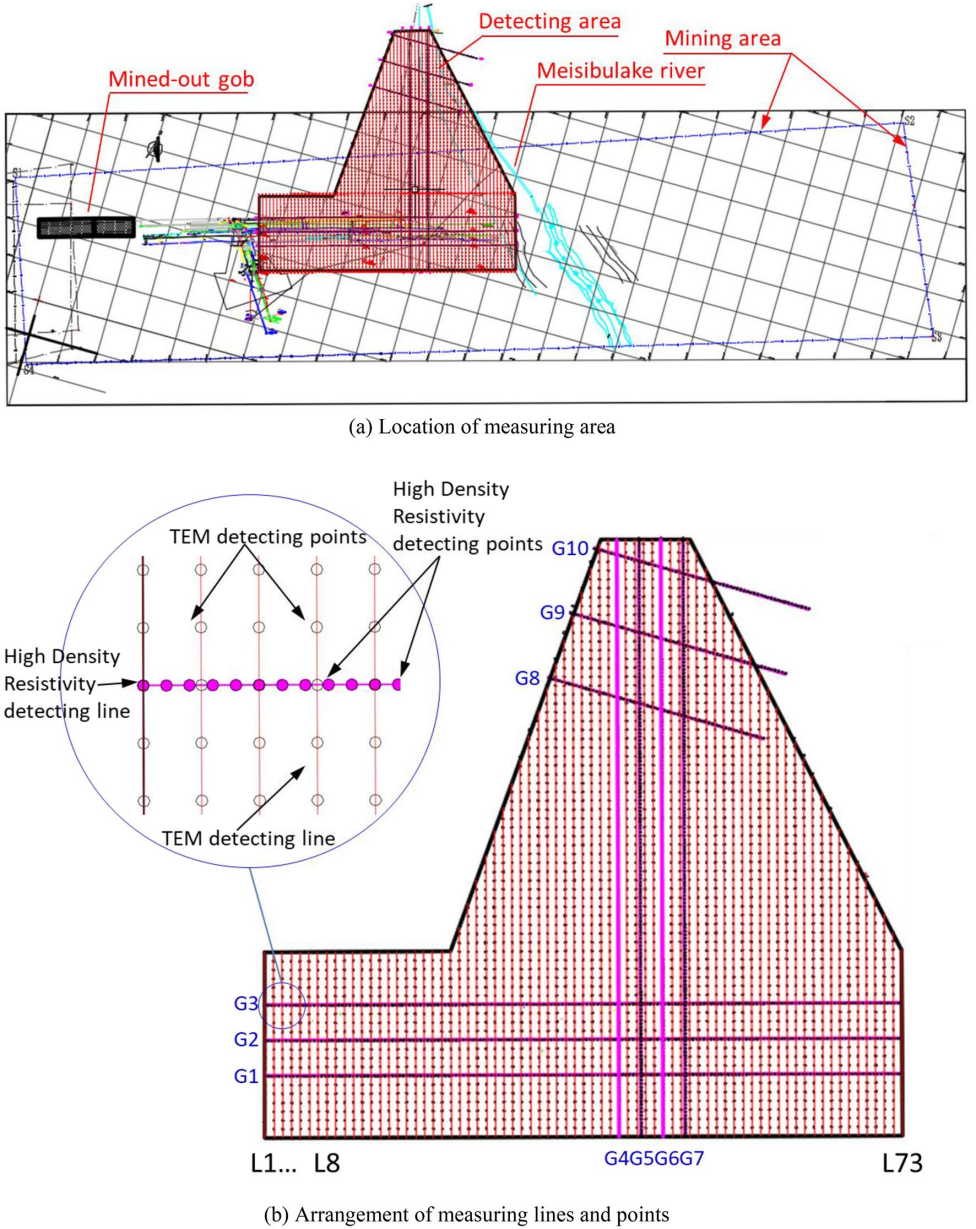
Measuring area. (**a**) Location of measuring area (**b**) Arrangement of measuring lines and points.

**Figure 3:**
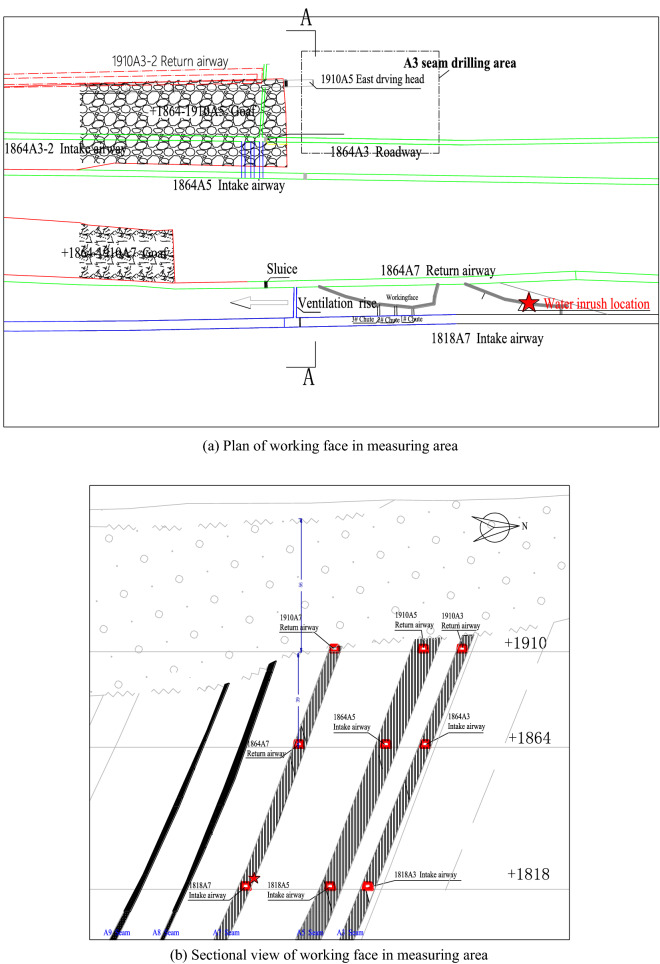
The plan and sectional view of working face in measuring area. (**a**) Plan of working face in measuring area, (**b**) Sectional view of working face in measuring area.

The working scope of geophysical exploration is 1.17 km^2^. In Fig. 2, the red polygon is the geophysical exploration area, and the black shadow area is abandoned coal mine. The plan and section of the working face of water inrush accident are shown in the Fig. 3.

### Geological structure

Most of the coal seams in this mine are buried below the local erosion datum. According to drilling information, the bedrock fissure aquifer of the roof and floor is mainly composed of hard and brittle medium sandstone, coarse sandstone, gravel coarse sandstone and conglomerate, which have certain pores and fissures. The aquifer is recharged by surface water and Quaternary phreatic water and stores a certain amount of groundwater. The primary water sources of the mine field are described as follows:

1. Coal seam water and roof and floor bedrock fissure water
  By means of drilling, simple hydrological observation and pumping test, it has been found that there are aquifers in the roof and floor. The hydraulic connection between the aquifers is extremely weak due to the influence of a quiclude. Judging from the current exploration situation, the coal seam water and roof and floor bedrock fissure water is the main source of this coal mine.
2. Quaternary rushed-diluvial gravel stone layer
  The Quaternary (Q2gl and Q3pl) sand and gravel layers with thickness from 0 to 60 m are inuniformly distributed on the surface of the mine field, generally thin in the west and thick in the east. The mine filed is recharged by surface water and H1 (Quaternary Holocene (Q4al + PL) alluvial sand-gravel aquifer) aquifer. When mining the shallow coal seam, the ground water in the Quaternary gravel stone layer would flow into the coal mine through water-conducted fissures in the overlying strata.
3. Surface water
  Meisibulake River flows through the mine field, from north to south, which is the only water source. Since the overlying strata are mostly composed of weak mudstone, carbonaceous mudstone and argillaceous siltstone, when the working face pass through the mine field, the surface water would flow into the coal mine through caving zone and water-conducted fissures.
4. Accumulated water in abandoned mines
  There is a certain amount of accumulated water in the abandoned mines, but the water volume is not clear. And when the working face enters the area around the abandoned mine, it may become the water source of the water inrush accident.

## Methods

### TEM

The TEM uses the ungrounded loop arranged on the surface to transmit a pulsed magnetic field to the underground space with a pulsed current to stimulate the secondary current induced by the underground conductive medium, and to collect the response of the secondary pulsed magnetic field during the pulse interval. When the stable current in the loop is suddenly cut off, the current in the transmission loop changes abruptly, and a vortex alternating electromagnetic field is generated in the conductors around it. The secondary field is an unstable magnetic field caused by the eddy current of different underground conductive media excited by the primary field. Under certain geological conditions, by analyzing the attenuation characteristics of the secondary field, the distribution location, development scale and occurrence electricity of the underground geological body can be inferred^21^.

The guiding concept of selecting TEM instrument is to improve the signal-to-noise ratio and resolution of collected data. V8 electrical workstation produced by Canadian Phoenix Company is used as the instrument ^22^. And the main performance parameters are shown in Table 1.

**Table 1:** Main performance parameters of TEM apparatus.

Parameters	Value	Parameters	Value
Maximum current output (A)	40	Sampling window	69 at most
Average output power (kw)	2.8	Synchronization method	Cable
Preamp gain (times)	1–10	Stacking fold	32–4096
Fixed gain (times)	3	Maximum sampling rate (μV)	2
Floating point gain (times)	2, 8, 32, 128	Minimum resolution (μV)	0.2

**Figure 4:**
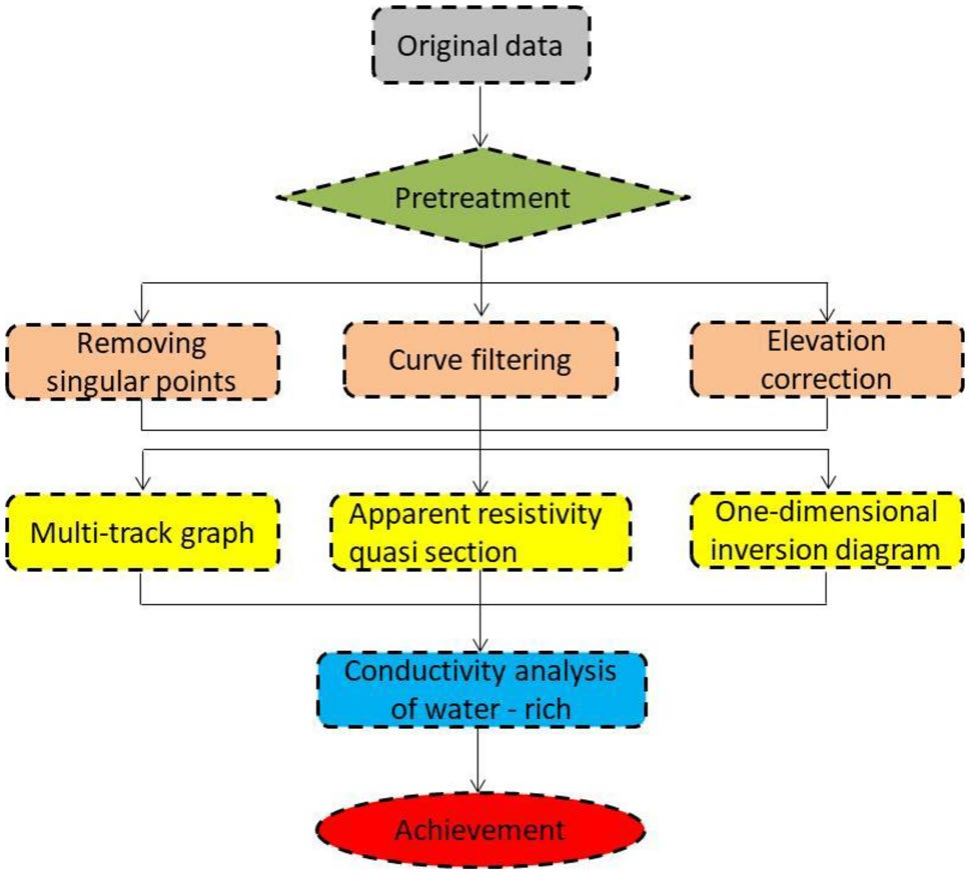
The flow chart of TEM data processing.

TEMPRO and IX1D transient electromagnetic processing software are used for the processing of data collected via TEM. Figure 4 shows the main process of data processing. The data from TEM is interpreted based on the induced voltage multi-channel section, resistivity quasi-section and resistivity bedding slice after data processing.

### HDRM

**Figure 5:**
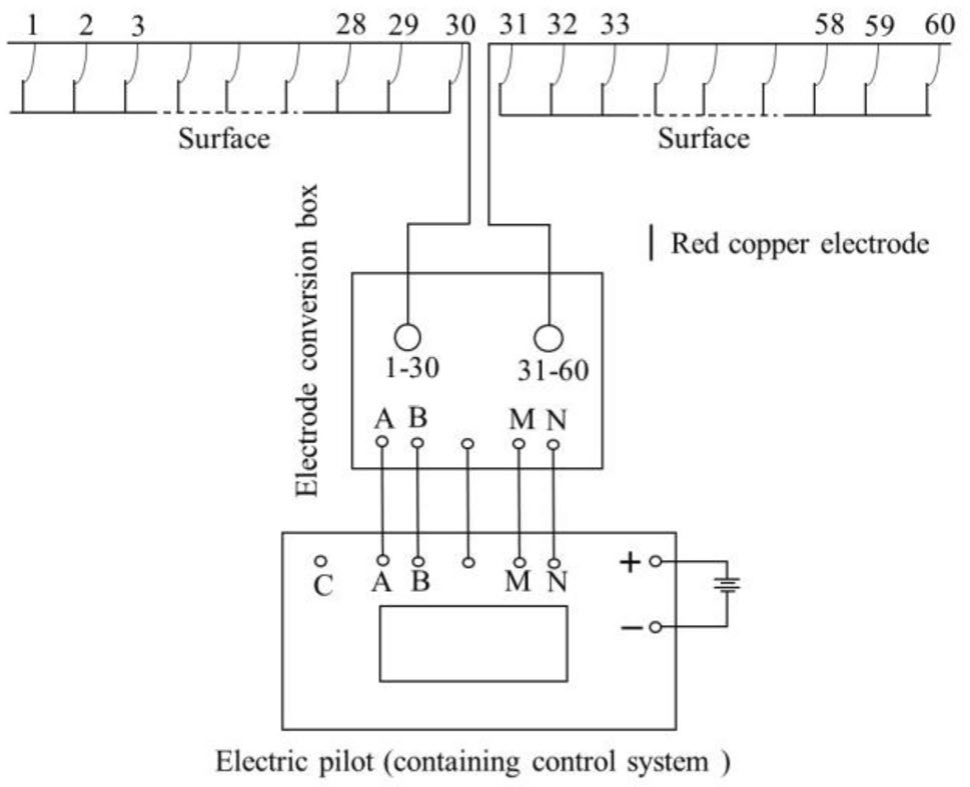
HDRM measuring system structure diagram^16^.

HDRM is an electrical exploration method to study the distribution of conducted current in the ground under the action of artificial stable current field on the basis of the difference of electrical conductivity between rock and soil. In the case of field measurement using HDRM, researchers simply place all electrodes (tens to hundreds) on each measuring point of the observation section, and then use program-controlled electrode conversion device and microcomputer engineering electric measuring instrument to achieve fast and automatic data acquisition. The distribution design is often used in the new generation of HDRM prospecting apparatuses. The distributed intelligent electrode is connected in series on the multi-core cable with randomly assigned addresses, which enables it to measure signals at any position, and realize rolling and continuous measurement of multi-channel and long profile, as shown in Fig. 5.

This project adopts the “E60DN ultra-high density electrical exploration system” developed by the Institute of Engineering and Technology of Jilin University, with 3 lithium ion power relay stations, 15 electrode switches (8 electrode switches/strings) and 120 stainless steel electrodes.

The system is the most advanced sounding instrument in China, and the main performance parameters are as follows: the receiving part has 10 channels which support resistivity and IP dual mode measurement, and the A/D converted digits are 24 bits; the input impedance is bigger than 150 MΩ; the measurement resolution is smaller than 30nV; the dynamic range is no less than 120 dB. The maximum output peak value is 400Vpp/1App, and the pulse type is square wave; For ulse length, the program control options ranging from 1 to 32 s are available.

**Figure 6:**
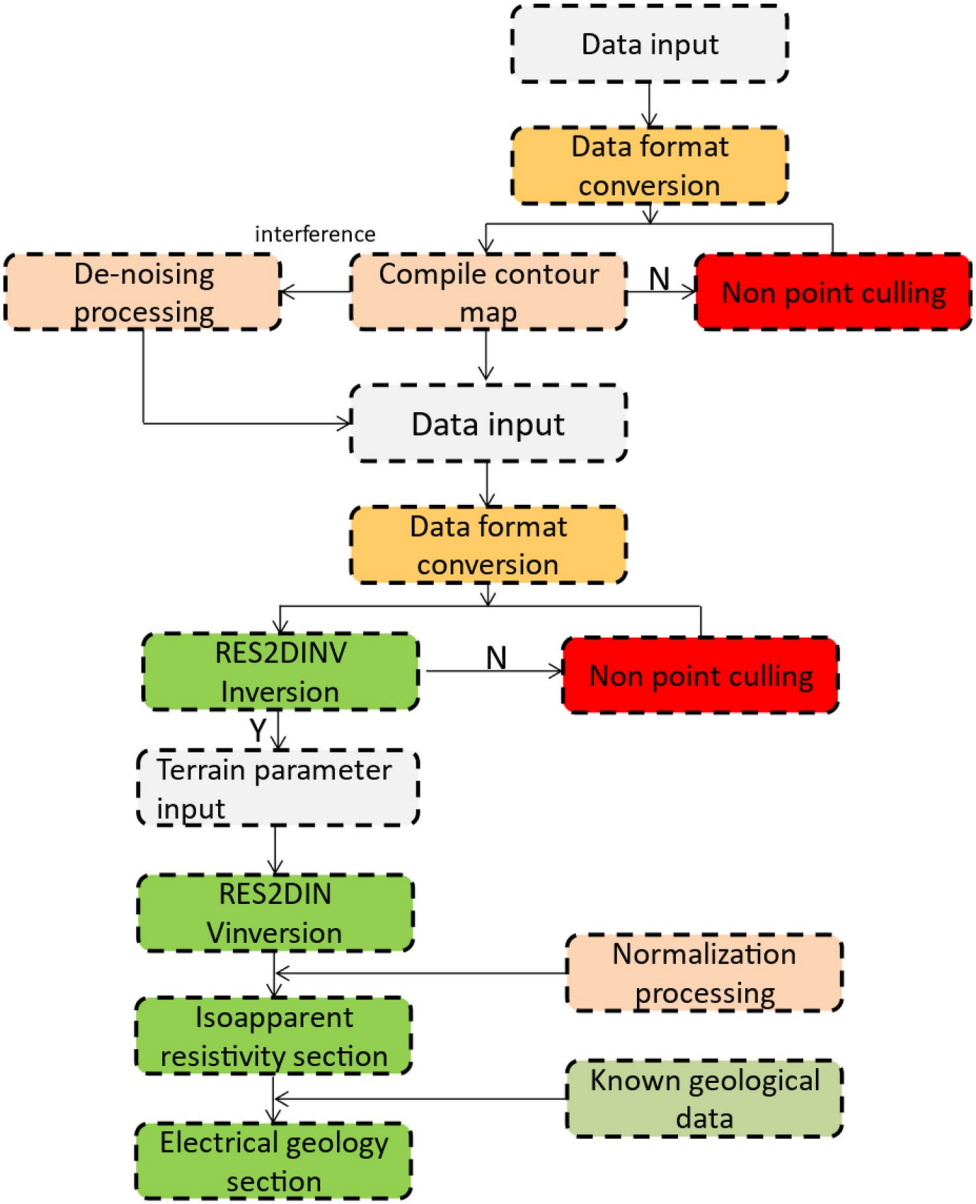
The flow chart of HDRM data processing.

The flow chart of HDRM data processing is shown in Fig. 6. The interpretation of HDRM is carried out synchronously with the data processing. Through the analytical study of the geophysical results of the known sections, the geophysical characteristics of the water-enriched areas are summarized, according to which the interpretation principle of geophysical anomaly is determined. On this basis, other geophysical profiles and anomaly areas are interpreted.

## Results and discussion

### Exploration results of TEM

First, according to the development position of the aquifer of the coal mine, the geophysical anomalies are interpreted in the profile. Then, these anomalies are combined by the horizontal slice map, and finally the shape, distribution range and spatial location of the water diversion channel and recharge water source can be inferred.

#### Interpretation results of typical longitudinal section

**Figure 7:**
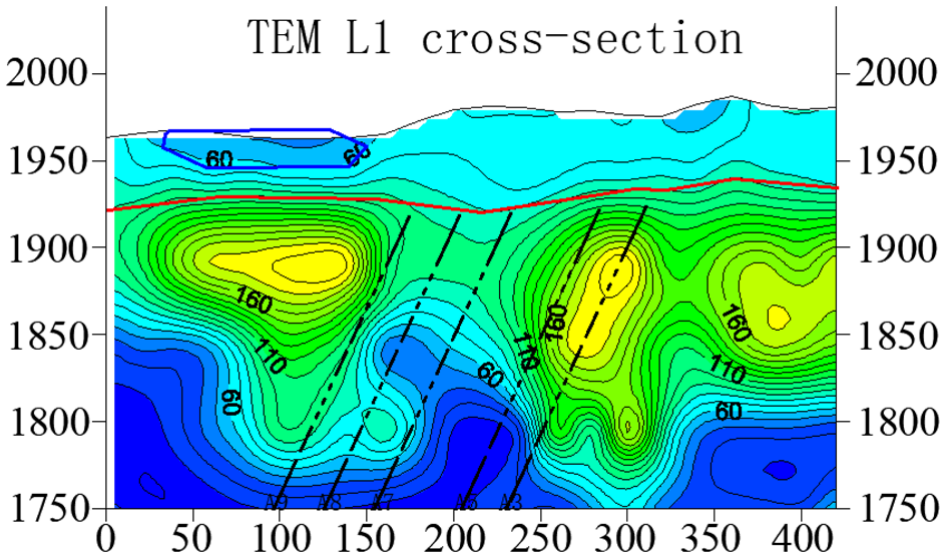
The resistivity profile of the TEM of L1.

**Figure 8:**
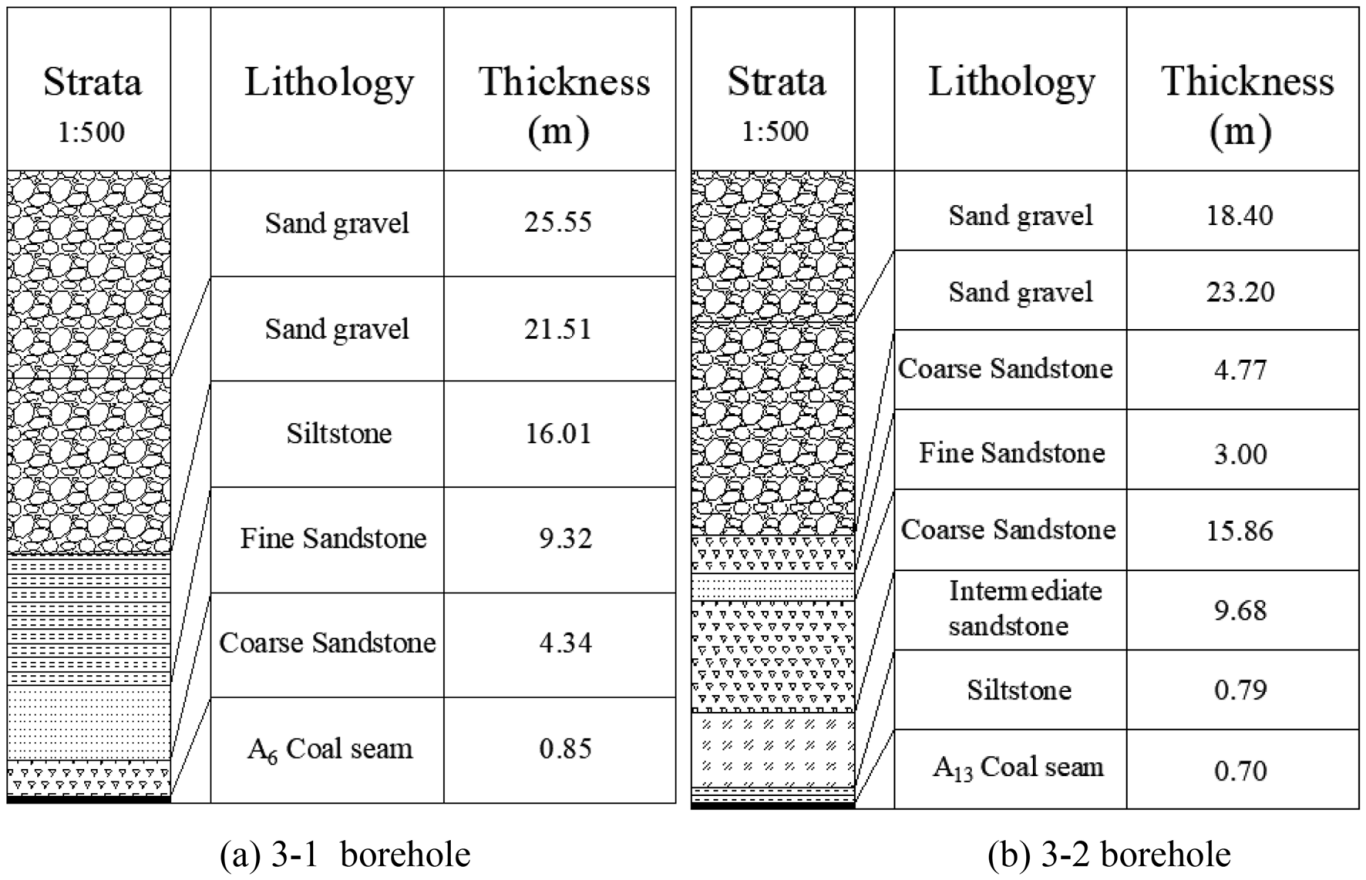
Drill column of geological prospecting boreholes. (**a**) 3–1 borehole, (**b**) 3–2 borehole.

Figure 7 shows resistivity variation of section L1. From the profile, it can be seen that the resistivity of Quaternary is generally low and the bedrock interface is obvious. This exploration line coincides with the Line 3 of the earlier geological prospecting section. And the drill column of geological prospecting boreholes 3–1 and 3–2 is shown in Fig. 8. The thickness of Quaternary inferred by geophysical exploration is consistent with the geological conditions revealed by boreholes, and the thickness is about 50–80 m. In the 25–150 m range of exploration station, the resistivity of Quaternary strata is smaller than 60 Ω•m, which is enriched by water and tends to connect with bedrock. Thus, it is the direct recharge source of water content in bedrock. The resistivity of the large mileage direction is higher than that of the small mileage direction, so it can be inferred that the small mileage range of the exploration line is enriched by water.

**Figure 9:**
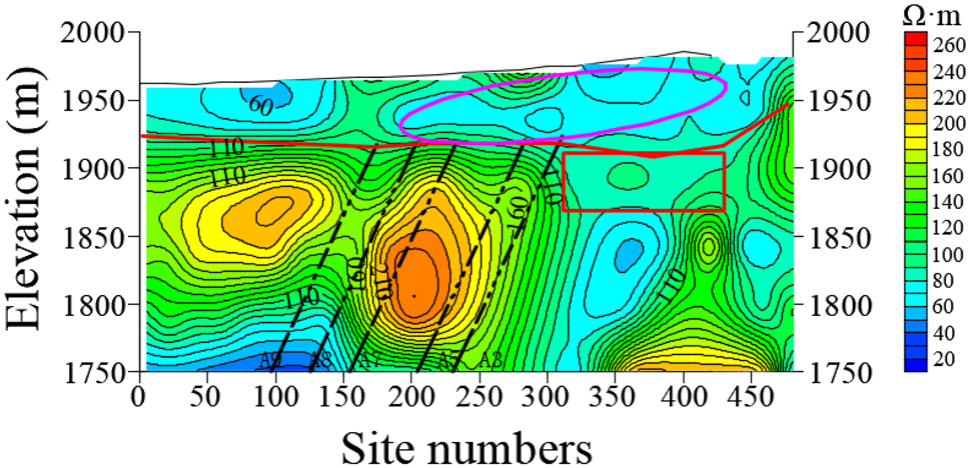
The resistivity profile of the TEM of L23.

**Figure 10:**
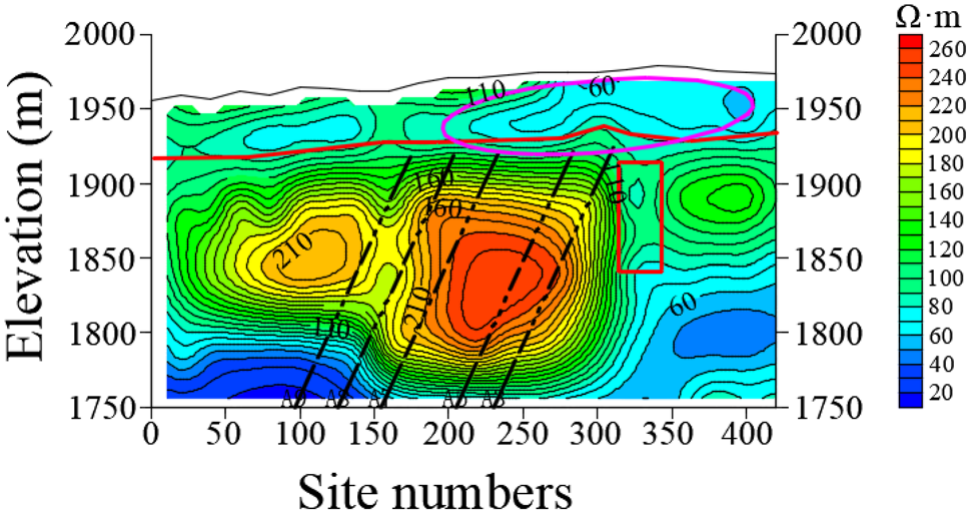
The resistivity profile of the TEM of L13.

Figures 9 and 10 show the resistivity variation of L23 and L13 sections, respectively. The resistivity of bedrock in the area delineated by magenta line of station 300–450 m is low, which indicates that the water enrichment of bedrock is obviously recharged by Quaternary aquifer. The Quaternary in this area is directly recharged by surface water, rainwater and snowmelt water. By comparing L23 and L13 sections, the Quaternary thickness of L23 is thicker than that of the L13. The elevation of L23 and L24 is 1750 m, and the anomaly depth of low resistance in the 0–150 m area is larger.

**Figure 11:**
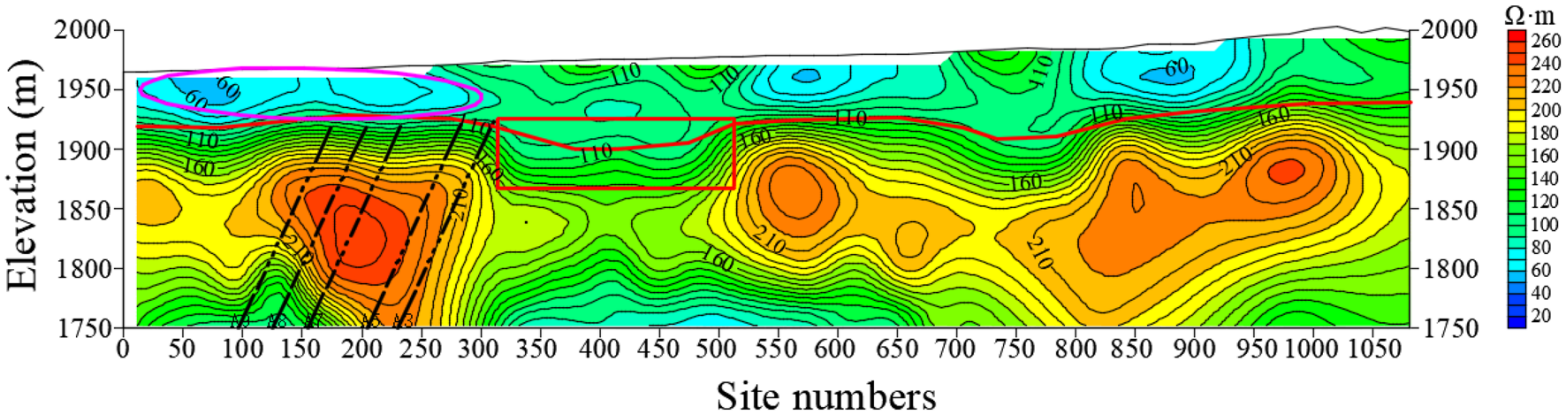
The resistivity profile of the TEM of L34.

Figure 11 shows the resistivity variation of L34 section. On the whole, the resistivity of Quaternary is generally low, basically less than 100 Ω•m, and less than 60 Ω•m in some places, indicating that the Quaternary strata are enriched by water but unevenly distributed. The resistivity of the Quaternary above the coal seam is lower than that of other places, which shows that the water enrichment is strong. Thus, when the working face passes through this place, the goaf would be directly recharged by the Quaternary aquifer, easily leading to a water inrush accident. The resistivity of the bedrock in the magenta line of the station 300–500 m section is low, indicating that the water enrichment of the bedrock is obviously recharged by the Quaternary aquifer.

**Figure 12:**
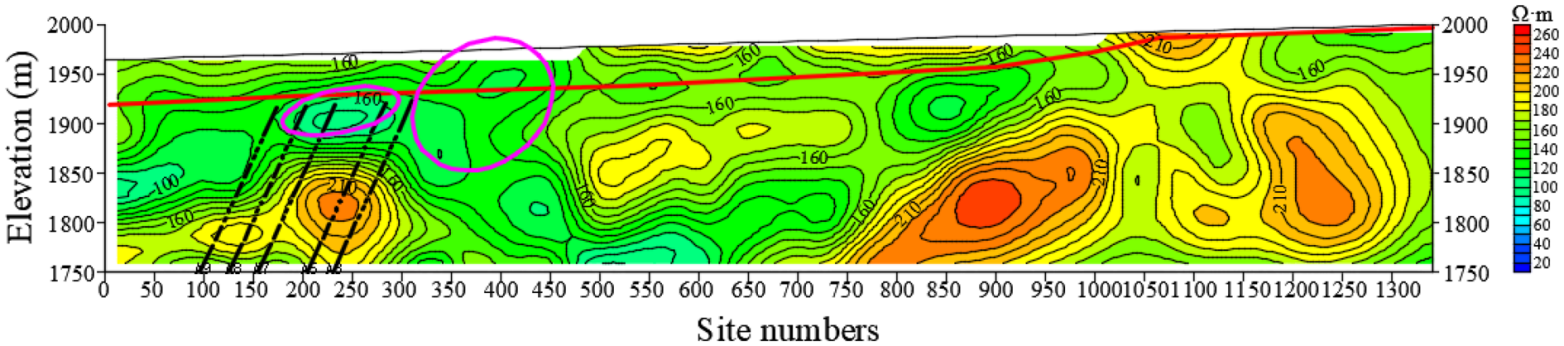
The resistivity profile of the TEM of L44.

A total of 73 section lines are drawn. Taking section L44 as an example (Fig. 12), the abscissa is the geophysical station number, and the ordinate is the elevation. The red virtual line along the direction of the exploration line is the bedrock interface inferred by geophysical prospecting, and the above is the Quaternary strata; the black virtual line is position of A3, A5, A7, A8, A9 coal seams on the profile inferred by geophysical prospecting.

The overall resistivity of the section is higher, but the Quaternary strata delineated in the 0–500 m section of the station are obviously lower, indicating that the strata are enriched by water, from which it can be presumed that there is a water supply relationship with the coal measure strata. There is an obvious low-resistivity area between the surface and the bedrock surface of the 500–750 m section, showing a channel shape. The Quaternary low resistivity anomaly between the station 750–1050 m is obvious, and there is an obvious water supply relationship with the underlying strata. The roof water inrush accident occurred in the + 1818 m A7 east working face, which is consistent with the anomaly low-resistance area delineated by the red oval line on the profile, and it is inferred that the water inrush source is the Quaternary aquifer. Therefore, the Quaternary is obviously enriched by water and is supplied by surface water.

**Figure 13:**
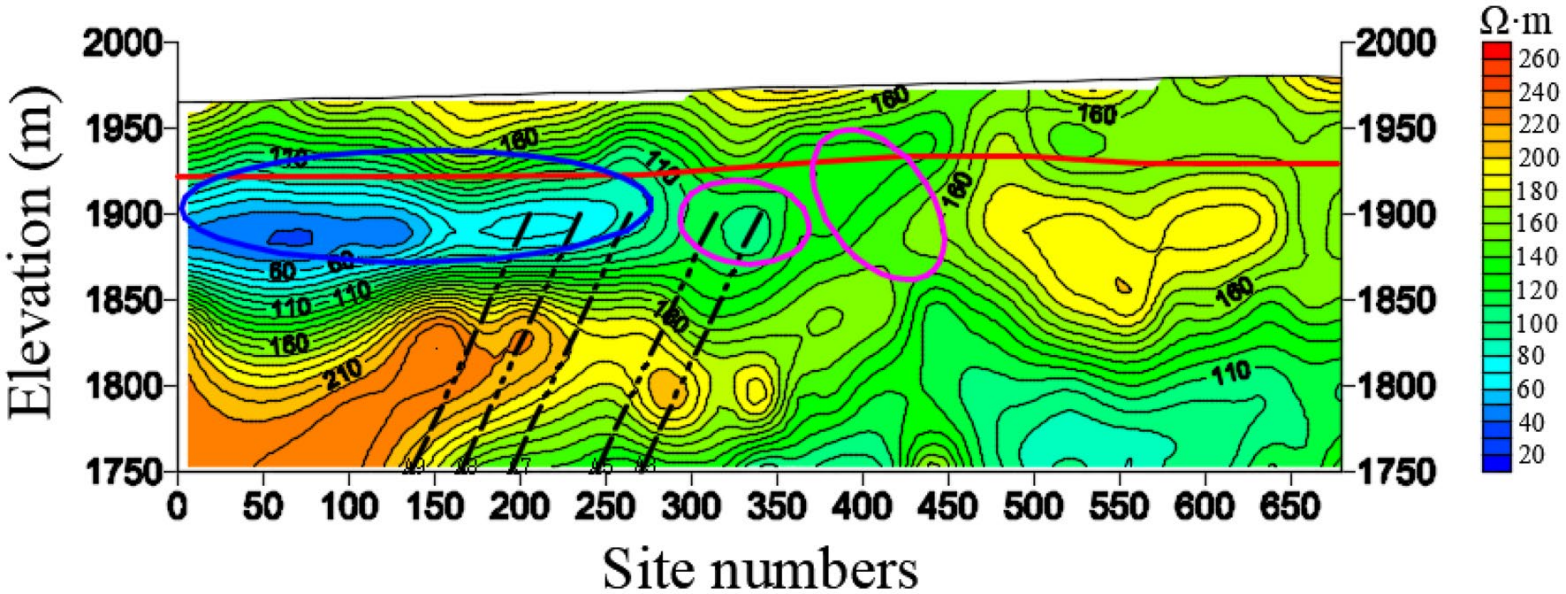
The resistivity profile of the TEM of L66.

Figure 13 shows resistivity variation of section L66. From the profile, it can be seen that in the 0–280 m range of exploration station, the resistivity of Quaternary strata is less than 60 Ω•m, which is enriched by water. The Quaternary thickness of the exploration line gradually thickens from large mileage to small mileage, and the elevation of bedrock interface becomes smaller, which creates conditions for the formation of local strong aquifer in Quaternary. The formation of strong aquifer brings threats to safety mining. The aquifer is also shown on the L63-L73 sections and has a wide influence scope.

#### Interpretation results of typical cross section

**Figure 14:**
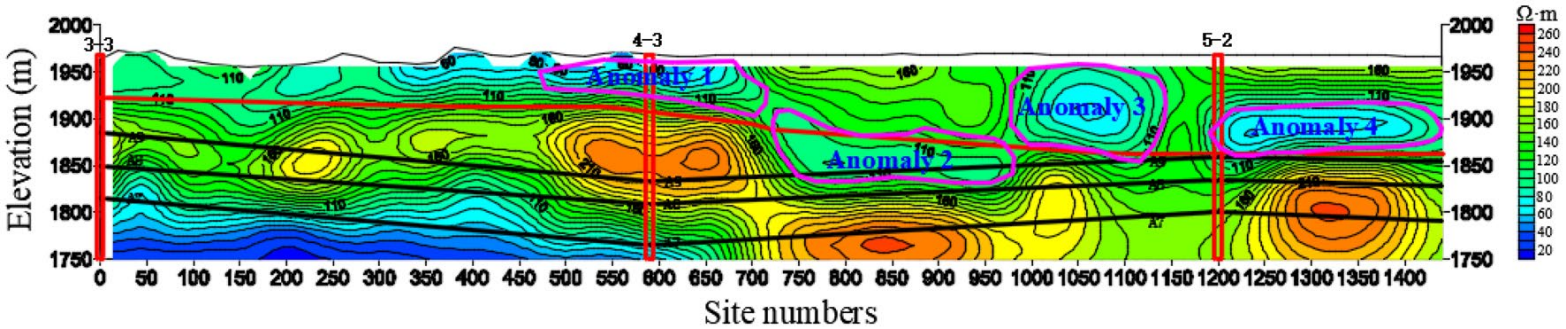
The resistivity profile of the TEM of 140 m mileage cross-section.

12 cross-sections are drawn according to the monitoring data. Taking the 140 m mileage cross-section as an example (Fig. 14), the abscissa is the geophysical station number, and the ordinate is the elevation. The red virtual line along the direction of the horizontal exploration line is the bedrock interface inferred by geophysical exploration, and the above is the Quaternary strata; the black virtual line is positions of A9, A8, A7 coal seams on the cross-section inferred by geophysical exploration. 3–3, 4–3 and 5–2 are the positions of geological prospecting boreholes on the figure.

It can be found that the Quaternary thickness of the exploration area is basically consistent with the previous borehole data. There is an obvious low resistivity anomaly area at the 700–1000 m station, the upper part of which is connected with the Quaternary and extends to the mileage of 1150 m. There is an obvious water-bearing anomaly in the Quaternary at the station 1200–1440 m, which is close to the coal seam and has no aquiclude, which poses a major potential hazard to mining safety in the future. The depth of the anomaly low-resistivity area in the section is 1750 m and the mileage is 0–550 m.

**Figure 15:**
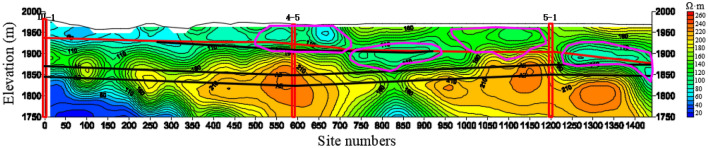
The resistivity profile of the TEM of 220 m mileage cross-section.

Figure 15 shows the resistivity variation of 220 m mileage cross-section, and III-1, 4–5 and 5–1 are the positions of geological exploration boreholes. The 220 m mileage cross-section is located at 80 m north of the 140 m mileage cross-section. From the resistivity map, it can be seen that the characteristics of the Quaternary are stable and the anomaly positions circled by the two cross-sections are also consistent.

**Figure 16:**
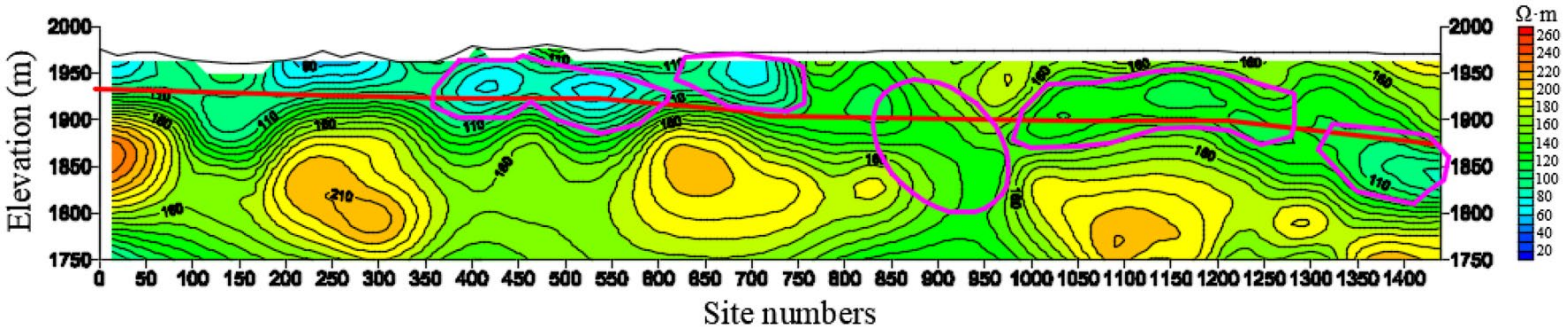
The resistivity profile of the TEM of 300 m mileage cross-section.

**Figure 17:**
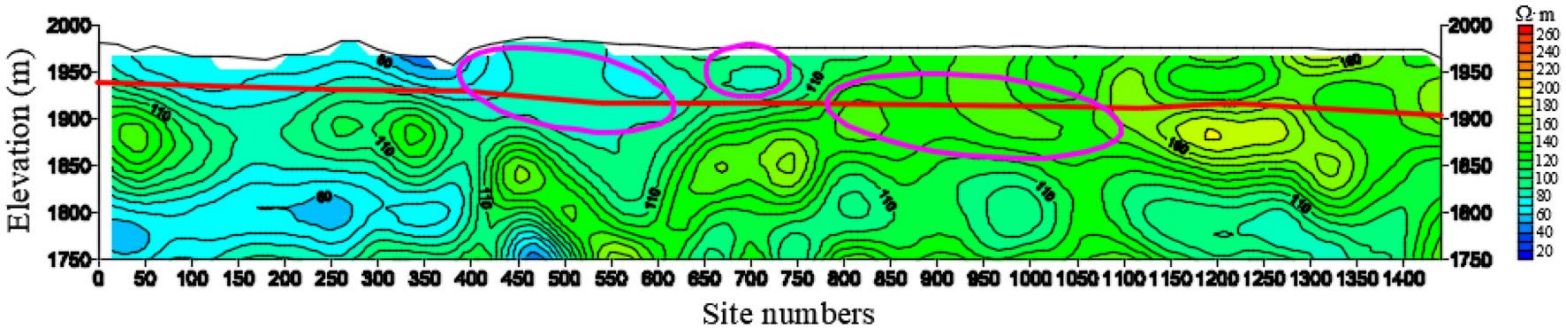
The resistivity profile of the TEM of 420 m mileage cross-section.

Figures 16 and 17 show the resistivity variation of 300 and 420 mileage cross-sections, respectively. From Fig. 16, there is an obvious low-resistivity area around 700–1100 m station, which is closely related to the coal-bearing series strata. From Fig. 17, the overall resistivity in this area is low and the Quaternary is enriched by water.

#### Interpretation results of horizontal slices

**Figure 18:**
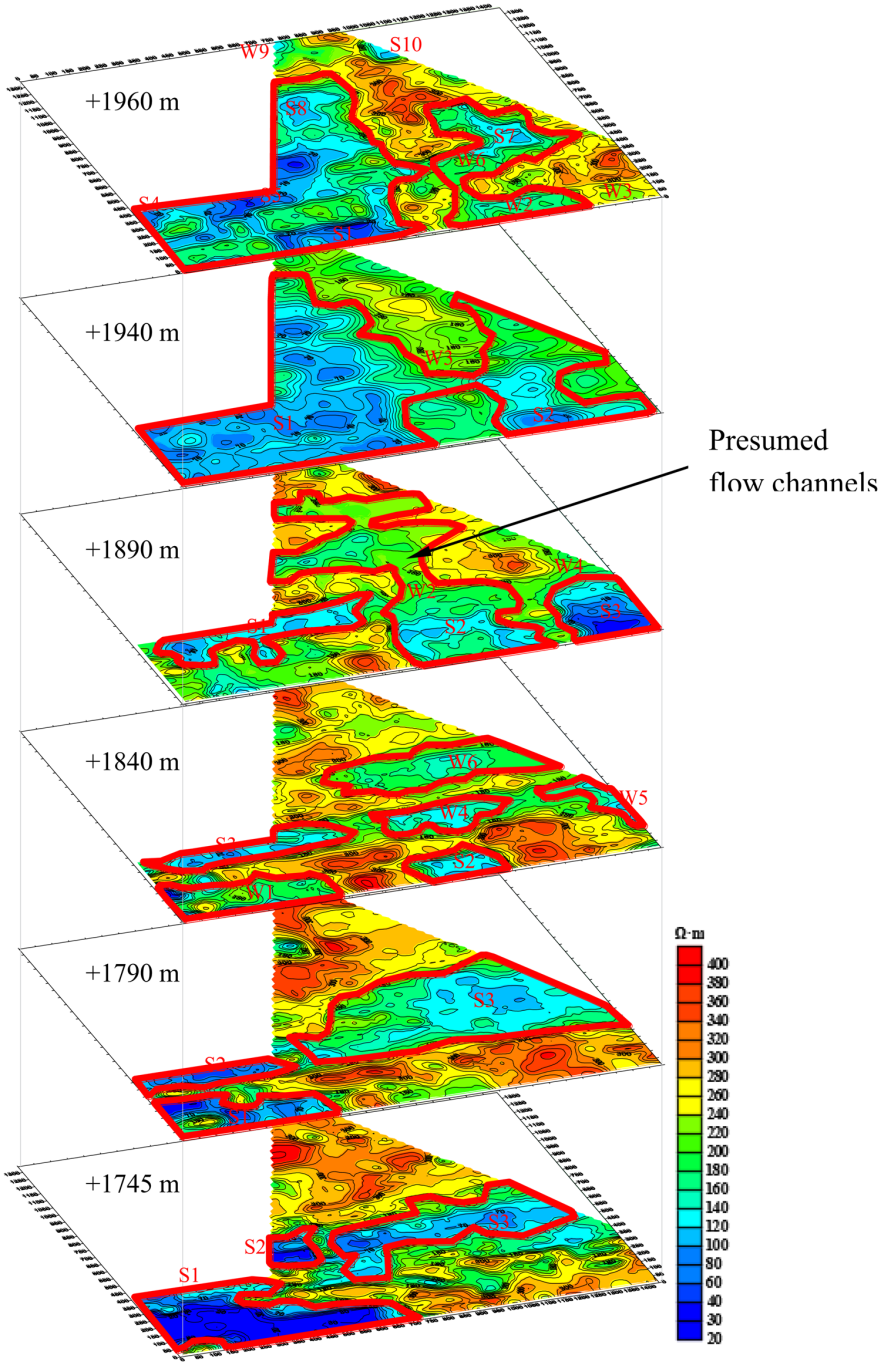
The resistivity profile of the TEM of horizontal slices (**a**) + 1960 m, (**b**) + 1940 m, (**c**) + 1890 m, (**d**) + 1840 m, (**e**) + 1790 m, (**f**) + 1745 m.

To easily understand the position of water-rich areas and identify the potential water-conducting pathways, a 3D scenario containing the detection results of the horizontal slices of + 1960 m, + 1940 m, + 1890 m, + 1840 m, + 1790 m and + 1745 m by TEM is shown in Fig. 18. The area circled in red is inferred to be enriched by water according to geophysical exploration.

The water-enriched area is divided into strong water-enriched area and weak water-enriched area according the resistivity value. The resistivity value of strong water-enriched area is lower than 100, while the resistivity value of weak water-enriched area is between 100 and 180. In the figure, prefix “S-” represents “strong water-enriched area”, and prefix “W-” represents “weak water-enriched area”.

10 anomaly areas are interpreted at + 1960 m level, all of which are Quaternary water-enriched areas, including 6 strong water-enriched areas and 4 weak water-enriched areas. Because it is near-surface stratum, it is greatly affected by the surface water, and water enrichment is extremely unstable.

The groundwater level is + 1944.62 m, which belongs to water weak-enriched aquifer. And it is in good agreement with the above geological data. At + 1940 m level, there are different degrees of water-enriched in the whole area near the elevation, which interprets 2 large-range strong water-enriched areas and 1 weak water-enriched area.

At + 1890 m level, there are 3 strong water-enriched areas and 2 weak water-enriched areas. Because S1 and W4 are close to the coal seam, they should be taken seriously into consideration. Since there were mining activities passed through S2 and S3 before, it is inferred that they are mined-out water-logged zones with large amount of water accumulation. Besides, the Quaternary cover in this area is extremely thick, the Quaternary cover thickness gradually thickens from north to south in the magenta shadow area. The Quaternary aquifer directly covers the Jurassic coal measure strata, and the bedrock interface is also high in the north and low in the south. This creates geological conditions for the flow of groundwater to the south. It is inferred that there is a stable water runoff channel at this position, and the bottom boundary of the channel approaches the bedrock interface. From the anomaly development and the water-enriched changes of the above strata, the water in the channel is recharged by the water in the Quaternary aquifer, while the Quaternary aquifer is directly recharged by surface water, river water and snowmelt water. This also leads to the long-term stability of the amount of water in the runoff channel.

The elevation of + 1840 m, + 1790 m and + 1745 m reflects the change of water-enriched characteristic of bedrock, and the locations and development of several anomalies are very similar. With the decrease of elevation, the resistivity of anomaly display gradually becomes smaller. It is speculated that due to the uneven distribution of water enrichment, the more downward the water enrichment is, the stronger the water enrichment is, and the water-bearing range is more concentrated. The resistivity value in the figure is only a parameter to judge the water enrichment of the formation, and it does not mean that the smaller the resistivity value is, the greater the water content is, that is, it only reflects the change of the water enrichment to some extent. The whole circled anomaly areas need to be taken into account. According to the overall analysis, the Quaternary overburden layer in this area is extremely thick, which is greatly affected by surface water, and has strong water-enriched and water-conducting properties.

### Exploration results of HDRM

#### Interpretation results of exploration line along roadway

A total of 10 exploration lines are arranged with high density to further verify and supply the water content of shallow strata on the basis of TEM. The development location of the aquifer is determined based on geological data, and the geophysical anomalies can be interpreted in the profile. Finally, the shape, distribution range and spatial location of the water diversion channel and recharge water source can be inferred.

**Figure 19:**
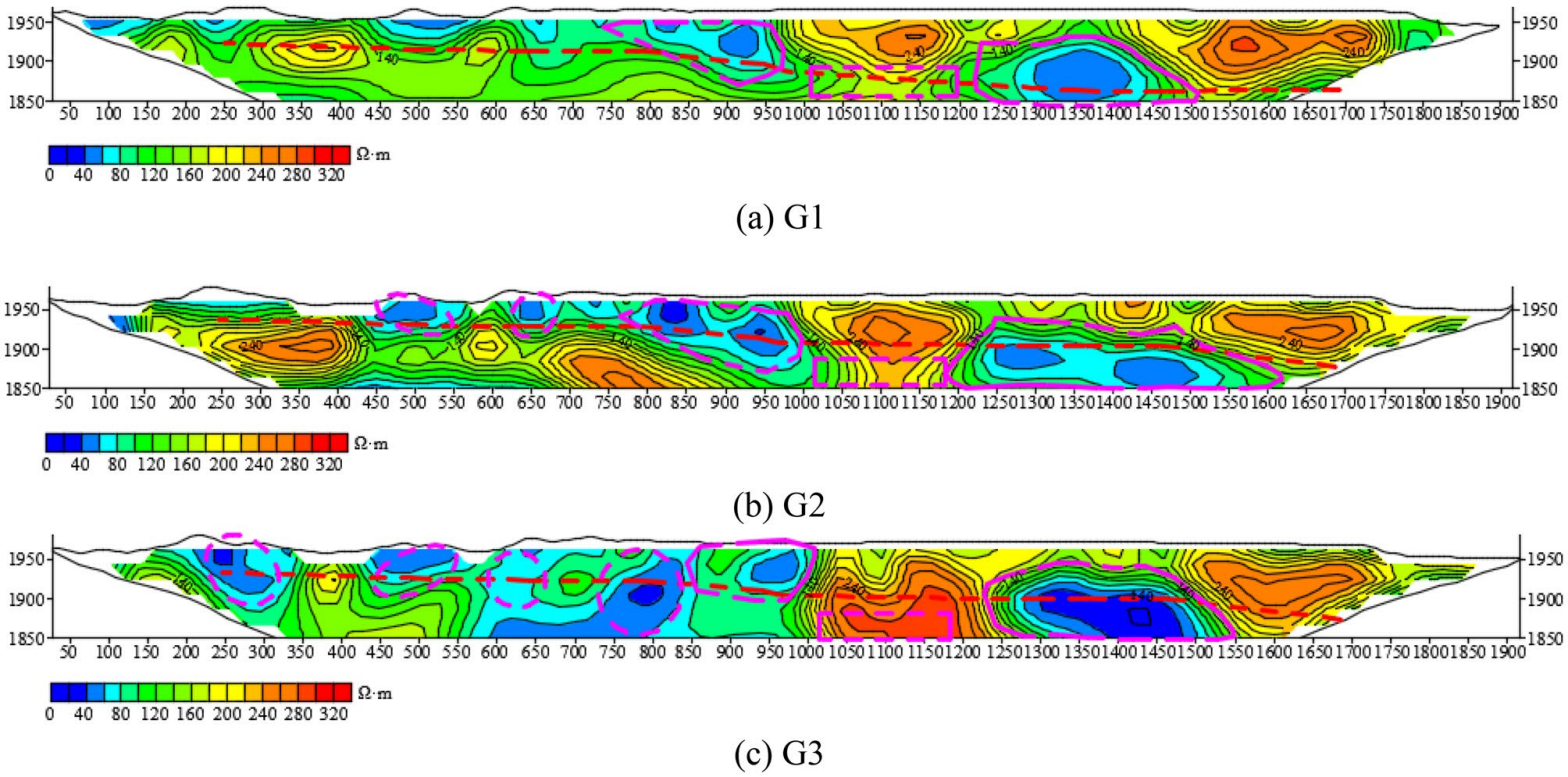
The resistivity profile of the HDRM of (**a**) G1, (**b**) G2, (**c**) G3.

Figure 19 shows the comparison of HDRM exploration lines of G1, G2 and G3 along the roadway. The magenta rectangular area in the profile of G1 exploration line is the mine water gushing section, which is also this exploration focus. It can be seen that, there is no obvious anomaly in G3 in this area, and the anomaly of G2 line begins to appear, and the anomaly of G1 line is obvious and tends to be connected with the left and right sides. There is an obvious low resistance anomaly on the right side of the water gushing section at the station 1250–1550 m, and there is also an obvious low resistance anomaly on the left side of the water gushing section at the station 800–900 m. Both are all enriched by water. The two anomalies have obvious responses on the three lines, and the circled anomaly positions are consistent, that is, the measurement results are consistent and reliable.

#### Interpretation results of vertical roadway exploration line

**Figure 20:**
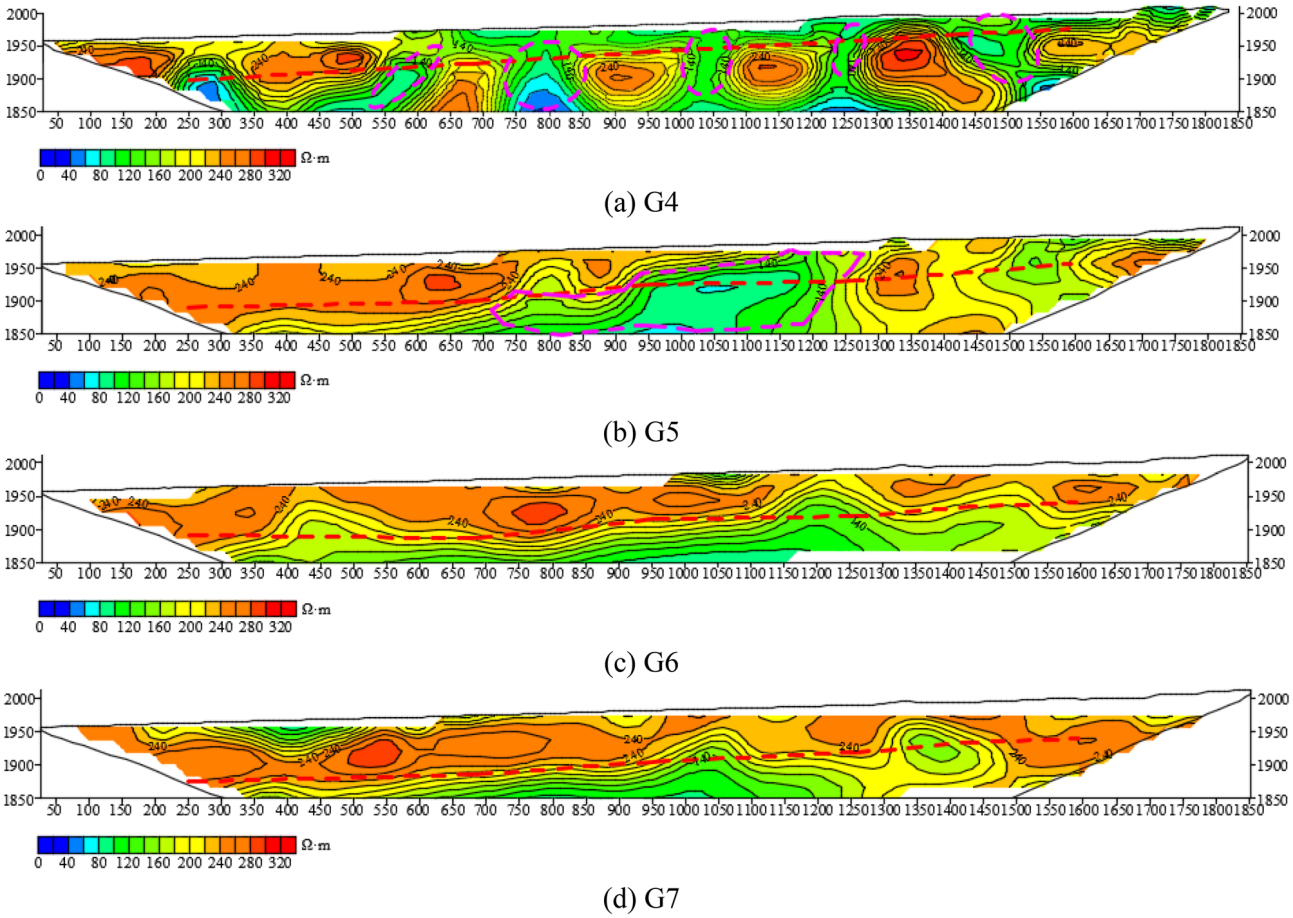
The resistivity profile of the HDRM of (**a**) G4, (**b**) G5, (**c**) G6, (**d**) G7.

Figure 20 shows four HDRM exploration lines arranged in the vertical roadway, numbered G4, G5, G6 and G7, respectively. As can be seen from the G4 profile, the Quaternary of the G4 exploration line is relatively enriched by water, and the Quaternary is connected with the bedrock, and five anomaly areas are circled. A wide-range anomaly area is circled on G5 exploration line, and anomalies in other positions are not obvious. No obvious anomaly areas are found in G6 and G7 exploration lines.

#### Interpretation results of exploration lines in the north of the exploration area

**Figure 21:**
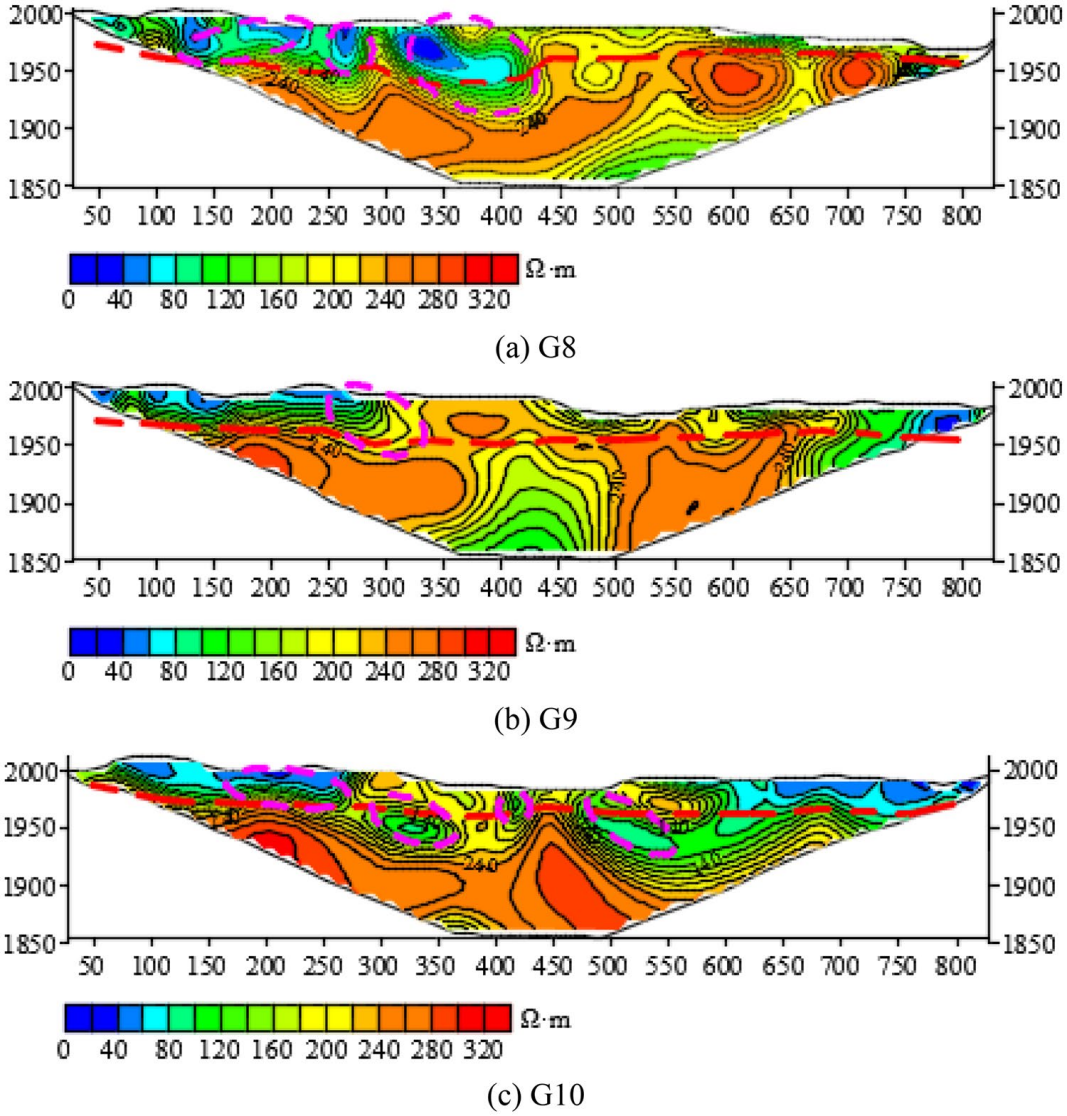
The resistivity profile of the HDRM of (**a**) G8, (**b**) G9, (**c**) G10.

Figure 21 shows the comparison of the resistivity isoline of G8, G9 and G10 exploration lines in the north of the exploration area. G10 is the northernmost exploration line, and G9 is located in the south of G10, and the vertical distance is 154 m; G8 is in the south of G9, and the vertical distance is 155 m. G9 and G10 cross the Meisibulake River, and G8 is located on the west side of the Meisibulake River.

On the profile of the resistivity isoline, the G8 exploration line delineates three anomaly areas, all of which are close to each other and influence each other.

One anomaly area is circled on G9. 4 anomaly areas are circled on G10. The anomaly areas are sporadically distributed, and the resistivity is medium to low, which indicates low water-richness.

As can be seen from the comparison, the Quaternary becomes increasingly thinner toward the north, and its water enrichment is getting increasingly weaker.

### Comparison of TEM and HDRM results

**Figure 22:**
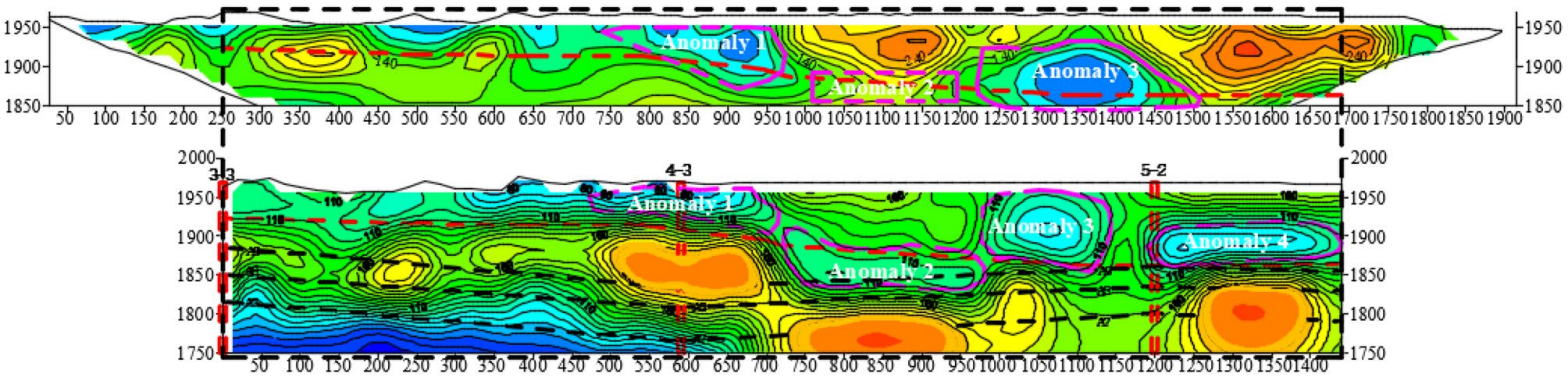
Comparison of resistivity profile of the TEM of 140 m mileage cross-section and HDRM of G1.

The 140 m cross-section of the TEM coincides with the G1 exploration line of the HDRM. Due to the special working mode, HDRM has a longer range of exploration line than that of TEM. According to the design and actual working conditions, the 0 mileage position of the transient electromagnetic mileage 140 m cross-section corresponds to the 250 m position of the HDRM G1 line section, as shown in Fig. 22. The horizontal red long dashed line in the figure is the inferred Quaternary lower boundary, the long black dashed line is the inferred coal bed line, and the vertical double red dashed line is the previous exploration drilling position.

On G1 line, 3 anomaly areas are interpreted through HDRM and 4 anomaly areas are interpreted through TEM. The corresponding relations are as follows: anomaly areas 1, 2 interpreted by HDRM correspond to anomaly areas 1, 2 interpreted by TEM, respectively, and their location and development scale are the same. The anomaly areas 3 and 4 interpreted by HDRM has large anomaly scale and strong water enrichment, corresponding to the position of anomaly areas 3 and 4 of TEM. The whole picture of transient anomaly area 4 cannot be detected because of the limited detection depth of HDRM. And the detection results of the water-bearing areas by the two methods are in good agreement with each other, which can confirm and complement each other, and the interpretation of the data is scientific and reasonable with high reliability. Besides, the detection depth of HDRM is larger than that of TEM.

## Conclusions

To conduct scientific treatment of water disasters in Mesbulake Coal Mine, the geophysical methods including TEM and HDR are used to quickly and effectively ascertain the cause of the roof water inrush and water supply channel. The principle, data processing method and main parameters of TEM and HDR method are introduced. In this geological exploration, 73 TEM survey lines and 10 HDR survey lines were arranged, and the exploration area was 1.17 km^2^. Based on the exploration results, the resistivity of water-bearing area is obviously low, which is generally lower than 60 Ω•m. The detection results of the water-bearing areas by the two methods are in good agreement with each other in terms of region and location. Besides, the detection depth of HDRM is larger than that of TEM. The following water-bearing strata situations are found out: 6 strong water-enriched areas and 3 weak water-enriched areas in + 1960 m elevation strata; 2 strong water-enriched areas and 1 weak water-enriched area in + 1940 m elevation strata; 3 strong water-enriched areas and 2 weak water-enriched areas in + 1890 m elevation strata; 2 strong water-enriched areas, 4 weak water-enriched areas and 2 water accumulation goaf in + 1840 m elevation strata; 3 strong water-enriched areas in + 1790 m elevation strata and 3 strong water-enriched areas in + 1745 m elevation strata. In addition, according to the geophysical interpretation results and geological data, the influence range of coal mine water inrush runoff channel is inferred and interpreted, and its location is preliminarily deduced.
